# The next era of renal radionuclide imaging: novel PET radiotracers

**DOI:** 10.1007/s00259-019-04359-8

**Published:** 2019-05-30

**Authors:** Rudolf A. Werner, Xinyu Chen, Constantin Lapa, Kazuhiro Koshino, Steven P. Rowe, Martin G. Pomper, Mehrbod S. Javadi, Takahiro Higuchi

**Affiliations:** 1 Department of Nuclear Medicine/Comprehensive Heart Failure Center, University of Wuerzburg, Oberduerrbacher Strasse 6, 97080 Wuerzburg, Germany; 2 Comprehensive Heart Failure Center, University of Wuerzburg, Wuerzburg, Germany; 3 Department of Nuclear Medicine, Hannover Medical School, Hannover, Germany; 4 Department of Biomedical Imaging, National Cardiovascular and Cerebral Center, Suita, Japan; 5 Division of Nuclear Medicine and Molecular Imaging, The Russell H. Morgan Department of Radiology and Radiological Science, Johns Hopkins University School of Medicine, Baltimore, MD USA; 6 The James Buchanan Brady Urological Institute and Department of Urology, Johns Hopkins University School of Medicine, Baltimore, MD USA; 7 Medicine, Dentistry and Pharmaceutical Sciences, Okayama University Graduate School, Okayama, Japan

**Keywords:** Kidney, Positron emission tomography, PET, [^18^F]Fluorodeoxysorbitol, GFR, ERPF

## Abstract

Although single-photon-emitting radiotracers have long been the standard for renal functional molecular imaging, recent years have seen the development of positron emission tomography (PET) agents for this application. We provide an overview of renal radionuclide PET radiotracers, in particular focusing on novel ^18^F-labelled and ^68^Ga-labelled agents. Several reported PET imaging probes allow assessment of glomerular filtration rate, such as [^68^Ga]ethylenediaminetetraacetic acid ([^68^Ga]EDTA), [^68^Ga]IRDye800-tilmanocept and 2-deoxy-2-[^18^F]fluorosorbitol ([^18^F]FDS)). The diagnostic performance of [^68^Ga]EDTA has already been demonstrated in a clinical trial. [^68^Ga]IRDye800-tilmanocept shows receptor-mediated binding to glomerular mesangial cells, which in turn may allow the monitoring of progression of diabetic nephropathy. [^18^F]FDS shows excellent kidney extraction and excretion in rats and, as has been shown in the first study in humans. Further, due to its simple one-step radiosynthesis via the most frequently used PET radiotracer 2-deoxy-2-[^18^F]fluoro-d-glucose, [^18^F]FDS could be available at nearly every PET centre. A new PET radiotracer has also been introduced for the effective assessment of plasma flow in the kidneys: Re(CO)_3_-*N*-([^18^F]fluoroethyl)iminodiacetic acid (Re(CO)_3_([^18^F]FEDA)). This compound demonstrates similar pharmacokinetic properties to its ^99m^Tc-labelled analogue [^99m^Tc](CO)_3_(FEDA). Thus, if there is a shortage of molybdenum-99, Re(CO)_3_([^18^F]FEDA would allow direct comparison with previous studies with ^99m^Tc. The PET radiotracers for renal imaging reviewed here allow thorough evaluation of kidney function, with the tremendous advantage of precise anatomical coregistration with simultaneously acquired CT images and rapid three-dimensional imaging capability.

## Introduction

Glomerular filtration rate (GFR) is defined as the rate of plasma flow through the glomerulus into the urinary space of the Bowman’s capsule and is the most suitable index and key indicator for renal function. A decrease in GFR to <60 ml/min/1.73 m^2^ for ≥3 months is a common criterion for defining chronic kidney disease and such a loss in GFR has been associated with a higher risk of all-cause and cardiovascular mortality [1]. In current clinical practice, GFR is estimated using serum creatinine; however, this method can be inaccurate and a separate assessment of individual renal function is not feasible [2, 3]. As the most accurate estimate of GFR, the exogenous marker inulin is considered as the gold standard for reliable assessment of GFR. However, because of technical difficulties and its high cost, it is seldom performed in clinical practice [4]. Blood clearance determined using [^51^Cr]ethylenediaminetetraacetic acid ([^51^Cr]EDTA) may be an attractive alternative, but information about split renal function cannot be obtained and the need for multiple blood collections limit its widespread use [5]. In this regard, a noninvasive metric, such as renal radionuclide imaging using single-photon-emitting [^99m^Tc]diethylenetriaminepentaacetic acid ([^99m^Tc]DTPA), is regularly employed in clinical routine, in particular as it offers the opportunity to determine split renal function in the context of GFR estimation [6]. This technique is quite well established in nuclear medicine centres to calculate renal blood flow and to evaluate unilateral kidney function, and its diagnostic performance has been proven in a variety of clinical settings [7–9]. However, procedures with [^99m^Tc]DTPA involving repeated blood and urinary measurements are a burden for both patients and personnel, and such lengthy procedures may also lead to noncompliance with procedural instructions, and flaws in sample collection [10].

As another marker of renal function, effective renal plasma flow (ERPF) can be derived from the clearance of para-aminohippuric acid infusion. Although it serves as a reference standard for ERPF assessment, this approach is not very well suited to clinical practice. Thus, in recent years, [^99m^Tc]mercaptoacetyltriglycine ([^99m^Tc]MAG3) has been routinely used to measure tubular extraction [11, 12]. However, Compton scatter and soft-tissue attenuation may reduce diagnostic accuracy and quantification reliability of these approaches. Of note, hybrid imaging using single-photon emission computed tomography/computed tomography (SPECT/CT) may overcome these hurdles, as it offers three-dimensional anatomical coregistration, but prolonged acquisition times and low spatiotemporal resolution still limit its potential for quantitative assessment [6, 13].

**Fig. 1:**
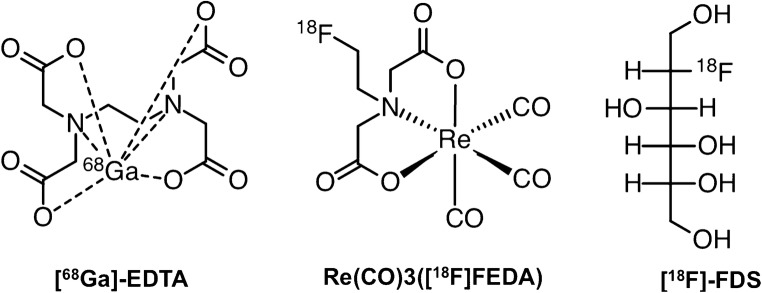
Chemical structure of [^68^Ga]ethylenediaminetetraacetic acid ([^68^Ga]EDTA), [^18^F]Re(CO)_3_-*N*-(fluoroethyl)iminodiacetic acid (Re(CO)_3_([^18^F]FEDA), and 2-deoxy-2-[^18^F]fluorosorbitol ([^18^F]FDS)

In contrast to conventional molecular imaging modalities, positron emission tomography (PET) radiotracers for renal functional assessment offer several key advantages, such as better spatiotemporal resolution, absolute camera-based quantification, and rapid three-dimensional imaging. In this regard, multiple renal PET radiotracers to assess renal function are currently under investigation, including [^68^Ga]EDTA, [^18^F]Re(CO)_3_-*N*-(fluoroethyl)iminodiacetic acid (Re(CO)_3_([^18^F]FEDA)), and 2-deoxy-2-[^18^F]fluorosorbitol ([^18^F]FDS) [14–17] (Fig. 1).

We provide an overview of these PET radionuclides for renal functional assessment, along with their underlying kinetic characteristics and potential uses in clinical routine.

## Quantitative data derived from a renal scan

The application of radionuclides to renal functional imaging is based on radiotracer kinetics and kinetic modelling, and thus renal molecular imaging allows reliable quantification. In this regard, various parameters can be derived from a renal scan and the quantitative information acquired includes, but is not limited to:

- *Renal perfusion*: Renal perfusion can be determined by visual and quantitative assessment of radiotracer transit after injection (through the abdominal artery and renal arteries) [18].
- *Relative renal uptake*: Relative renal uptake allows the assessment of differential renal function, for example by placing regions of interest (ROI) over the kidneys and measuring the integral of the counts in the ROIs after radiotracer injection [18]. Such split renal function assessments of the left and the right kidneys are of the utmost importance for living kidney donation, as disparity can significantly affect whether a donation can still be performed [19].
- *Maximal parenchymal activity (*T_*max*_*and* T_*1/2max*_*)*: *T*_max_ is defined as the time from injection to peak height of the renogram, while *T*_1/2max_ is the time for renal activity to decrease to 50% of its maximum value. The latter parameter is routinely used as a simple means for assessing renal obstruction. Although this parameter can be influenced by various factors (hydration status, radiopharmaceutical used, presence of a bladder catheter), a broad consensus exists that clearance with a *T*_1/2max_ of <10 min excludes the presence of obstruction [18, 20].
- *Camera-based clearance*: In contrast to plasma-based sample techniques, collection of blood and urine samples can be omitted for camera-based assessment of clearance. Tracer accumulation in the kidneys is determined shortly after injection of the radiotracer and divided by the counts injected. The percentage injected dose to the kidneys is converted to a clearance value (by comparison with a validated normogram) [18].

## Comparison of PET, single-photon planar imaging and SPECT in assessing renal function

Planar imaging techniques for renal radionuclide imaging have several drawbacks, including limited spatiotemporal resolution and missing anatomical information. Notably, hybrid imaging devices such as SPECT/CT scanners, allow three-dimensional assessments and anatomical coregistration, although these features are not commonly employed in clinical routine due to prolonged acquisition and low single-pass extraction, which are seen as major obstacles to reliable dynamic imaging. In addition, corrections for soft-tissue attenuation are required, e.g. by estimating renal depth or by applying an attenuation coefficient [21]. In contrast to SPECT, PET offers multiple advantages that can be considered key features for a more thorough evaluation of renal function. These include superior spatiotemporal resolution, absolute camera-based quantification approaches and multislice CT for anatomical co-registration. Most importantly, as compared to conventional single-photon scintigraphy/SPECT, count rates are significantly higher, which in turn may allow administration of much lower doses of radioactivity. For example, for a renal PET study with [^68^Ga]EDTA, 40 MBq is routinely administered. The effective dose from the PET component is 1.6 mSv and this equates to approximately 320 MBq of [^99m^Tc]DTPA [6, 15]. As a result, radiation exposure is minimized without sacrificing image quality.

**Fig. 2:**
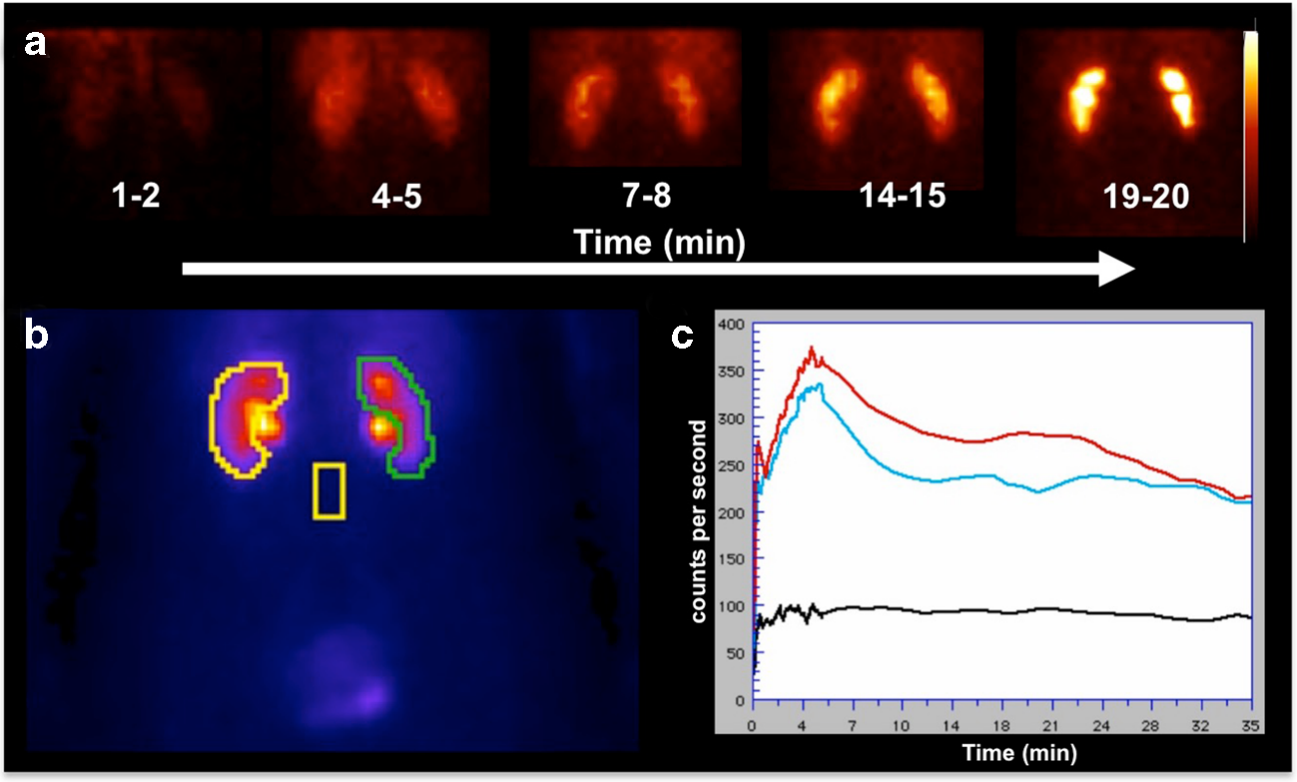
Right and left kidneys in a 52-year-old woman who underwent [^99m^Tc]diethylenetriaminepentaacetic acid ([^99m^Tc]DTPA) renal imaging evaluation for living kidney donation. **a** Dorsal planar images of both kidneys during imaging acquisition. **b** Regions of interest were placed over the entire kidneys on planar images. **c** Time–activity curves for the left kidney (*red*) and the right kidney (*blue*) show a normal renogram with a slightly better performance for the left kidney (GFR: left, 62 ml/min; right, 58 ml/min)

Consequently, the use of PET for renal imaging, including estimation of GFR, may improve the identification of structural abnormalities and quantification of obstructive processes in paediatric and adult subjects. In paediatric patients, the potentially lower radiation dose from PET radiotracers may be of particular importance, in particular if repeated renal studies are necessary [3, 15]. The introduction of time-of-flight technology, further improvements in detector technology and improved reconstructive algorithms may allow further decreases in the amount of administered activity [6]. Furthermore, the intrinsic ability of renal PET to provide tomographic images of the kidneys may allow elimination of background activity from surrounding organs, such as major vessels and the spleen [3]. Hence, time–activity curves may be generated from uptake exclusively in the kidneys and an automatically applied standardized uptake value threshold can be used to define activity in the cortex and collecting system [6]. This is in contrast to renal scintigraphy in which the ROI covers the entire kidney to generate the renogram (Fig. 2). Moreover, the accuracy of GFR obtained by SPECT agents such as [^99m^Tc]DTPA may also be adversely affected by pharmacokinetics. [^99m^Tc]DTPA has been postulated to be more strongly bound to plasma proteins than other radiotracers used for GFR assessment. Indeed, protein binding has been shown to vary by 10–13% and extracellular localization of DTPA may further adversely affect diagnostic accuracy [22]. However, novel PET radiotracers, such as [^68^Ga]EDTA, [^18^F]FDS and Re(CO)_3_([^18^F]FEDA), may have superior pharmacokinetic profiles, mainly due to low plasma protein binding and high metabolic stability (Fig. 1) [6, 14, 17].

## ^68^Ga-Labelled radiotracers for renal function imaging

### [^68^Ga]EDTA

Hofman and Hicks [6] were the first to report the use of the PET probe [^68^Ga]EDTA, which is almost exclusively excreted by glomerular filtration, for the assessment of renal function. After administration, the radiotracer initially concentrates in the blood pool, while the aorta (or heart) can potentially be used to provide input functions for kinetic analysis. Over time, activity increases in the renal cortex, followed by a gradual delineation of the renal parenchyma and transit of the activity into the collecting system, which can be observed 3–4 min after injection (p.i.). In a study of 31 patients, Hofman et al. [13] compared GFR estimates from [^68^Ga]EDTA PET/CT imaging and [^68^Ga]EDTA and [^51^Cr]EDTA plasma sampling. Three time-points were defined for PET quantification: an initial phase (1–2 min p.i.), a renal excretion phase (2–10 min p.i.) and a late phase (reflecting urinary excretion into the collecting system). GFR determined by the [^51^Cr]EDTA and [^68^Ga]EDTA plasma sampling methods showed an excellent correlation (Pearson correlation coefficient 0.94). GFR from the late phase PET quantification showed the strongest correlation with GFR from plasma sampling (correlation coefficient 0.9). The authors concluded that dynamic PET imaging using [^68^Ga]EDTA is as a noninvasive GFR estimation method with the additional advantage of hybrid renal imaging in the same study.

The same group also studied a small series of 11 patients with renal cell carcinoma who underwent both [^99m^Tc]DMSA (planar imaging, SPECT/CT) and [^68^Ga]EDTA PET/CT prior to stereotactic radiotherapy [6]. A significant discrepancy was noted between DMSA planar and SPECT/CT imaging (most probably owing to activity of adjacent overlying organs). Good agreement between SPECT/CT and [^68^Ga]EDTA PET during the excretory phase was found, whereas there was significant disagreement between the two imaging modalities in the early cortical phase, perhaps suggesting that [^68^Ga]EDTA may offer additional information in split renal functional assessment during the early phase of renal parenchymal transit [6].

### [^68^Ga]DTPA

Given that ^99m^Tc-labelled DTPA has been used for renal functional assessment over decades, Gundel et al. [23] investigated the renal PET probe [^68^Ga]DTPA in a head-to-head comparison with [^68^Ga]EDTA in vitro and in vivo in male Copenhagen rats. Only 30% of the injected [^68^Ga]DTPA activity was excreted via the kidneys (in contrast to almost 90% of [^68^Ga]EDTA activity). Of note, compared to measured inulin clearance, [^68^Ga]DTPA led to a marked underestimation of GFR by up to 80%. These findings are most likely explained by the strong binding of this radiotracer to plasma proteins [23]. Thus, compared to [^68^Ga]DTPA, [^68^Ga]EDTA demonstrates superior diagnostic performance in renal radionuclide PET imaging [6, 23].

### [^68^Ga]1,4,7-Triazacyclononane-1,4,7-triacetic acid ([^68^Ga]NOTA)

Lee et al. [24] evaluated ^68^Ga complexes (EDTA, DTPA and NOTA) and measured binding to serum and red blood cells, along with a head-to-head-comparison of GFR measurements with [^51^Cr]EDTA in mice. Notably, [^68^Ga]NOTA showed not only low binding to serum proteins, but also comparable GFR values to those obtained with the reference standard [^51^Cr]EDTA. Thus, [^68^Ga]NOTA may also be an attractive and easy-to-prepare renal PET agent [24]. However, compared to [^68^Ga]EDTA, feasibility studies in human subjects are still lacking [13]. In the 1960s, [^68^Ga]EDTA had already been tested in patients with glioblastoma using a positron scintillation camera with recording on Polaroid film [25]. Thus, compared to the recently introduced [^68^Ga]NOTA, this long experience with [^68^Ga]EDTA paved the way to the application of this radiotracer to renal PET imaging during the last decade [6].

### [^68^Ga]IRDye800-tilmanocept

Diabetic nephropathy is the leading cause of kidney failure and is characterized by progressive expansion of the mesangial matrix which finally occludes the glomerular capillaries [26]. Recently, Qin et al. [27] introduced the novel PET probe [^68^Ga]IRDye800-tilmanocept for the assessment of GFR in a rat study and the time–activity curves derived showed receptor-mediated renal accumulation with evidence of glomerular uptake. Further, histological examination investigating the colocalization of the tilmanocept receptor (CD206) and IRDye800-tilmanocept within the glomerulus confirmed mesangial cell accumulation of the radiotracer. In addition, diabetic and nondiabetic db/db mice underwent imaging with fluorescent-labelled [^99m^Tc]tilmanocept. The nondiabetic mice showed a single-phase time–activity curve with low bladder accumulation, while the diabetic mice showed a multi-phasic renal time–activity curve with high urinary bladder accumulation. Given the crucial role of the mesangial matrix in progression of diabetic nephropathy, the authors concluded that radiolabelled tilmanocept may be a novel receptor-based PET or SPECT imaging biomarker for monitoring progression of this glomerular disease [27].

## ^18^F-Labelled radiotracers for renal function imaging

^18^F-Labelled radiotracers have the advantage of lower positron energy with higher positron yield, which in turn opens the door to the injection of a considerably lower amount of activity without sacrificing image quality, and improves the contrast and noise characteristics of the images [28]. Moreover, ^18^F has a significantly longer half-life (110 min) than ^68^Ga (68 min), which allows delivery from central cyclotron facilities to smaller hospitals [29, 30]. Cyclotron production also allows nearly unlimited production of radionuclide in contrast to generator production. In this regard, an incremental cost-effectiveness ratio has already been proven for the most frequent PET radiotracer 2-deoxy-2-[^18^F]fluoro-d-glucose ([^18^F]FDG) [31]. Moreover, the longer half-life also allows more flexibility in study design, i.e. delayed imaging protocols that may provide further insight into radionuclide handling in the renal system. Of note, radiopharmaceuticals may also benefit from fluoride introduction, as the risk of metabolism at sensitive positions may be reduced [32]. In the following sections, we provide an overview of ^18^F-labelled radiotracers currently used for renal imaging.

### [^18^F]FDS

**Fig. 3:**
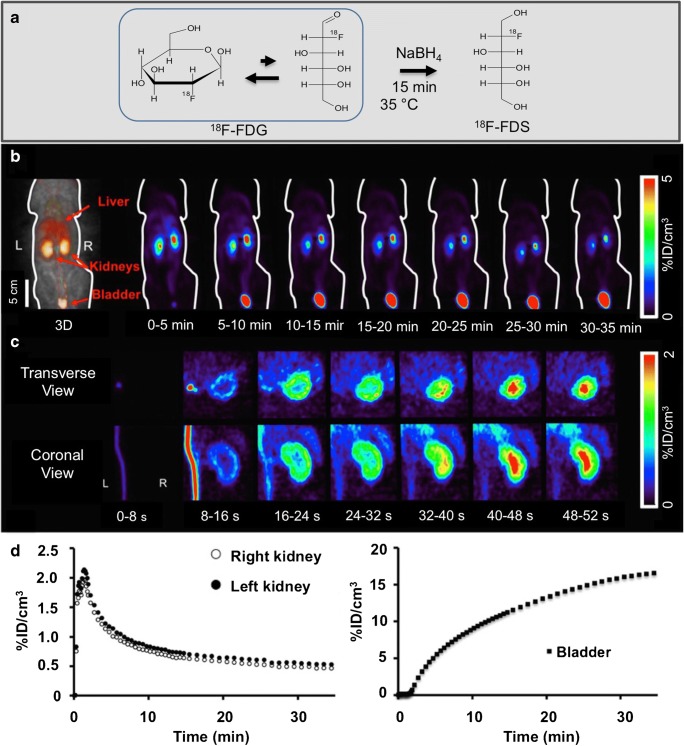
**a** [^18^F]Fluorodeoxysorbitol ([^18^F]FDS) radiotracer synthesis. [^18^F]FDS can be produced by methods adapted from Li et al. utilizing a simple one-step reduction from 2-deoxy-2-[^18^F]fluoro-d-glucose ([^18^F]FDG) [35]. **b**–**d** In vivo [^18^F]FDS PET imaging in healthy rats. **b** Whole-body dynamic coronal PET images show high tracer secretion exclusively via the kidneys and a time-dependent increase in bladder activity. **c** Dynamic transverse and coronal images of the right kidney show rapid tracer accumulation in the renal cortex and tracer transit into the collecting system. **d** Example time–activity curves for the kidneys (*left*) and bladder (*right*) assessed by dynamic PET imaging. Modified from Wakabayashi et al. [14]

Initially, [^18^F]FDS was developed for imaging in oncology and inflammatory diseases, in particular in clinical cases of known or suspected infections caused by Enterobacteriaceae [33, 34]. [^18^F]FDS can be easily synthesized by a simple one-step reduction of [^18^F]FDG, and thus [^18^F]FDS may be available in the near-term at many sites that have radiochemistry infrastructure (Fig. 3a) [35]. In early human studies, urinary clearance of sorbitol was found to be almost identical to that of inulin [36]. Given the underlying sorbitol structure of [^18^F]FDS, one could speculate that it inherits kinetic features almost identical to those of both sorbitol and inulin.

[^18^F]FDS was first investigated in healthy Wistar rats to determine its basic biodistribution properties as a renal PET probe, including clearance through the renal collecting system pathway, plasma protein binding and metabolic transformation. Dynamic PET images revealed high renal radiotracer excretion in healthy animals. After an initial blood flow phase through the inferior vena cava, gradual delineation of the renal cortex was observed and transit of the activity to the collecting system could be appreciated. In addition, a time-dependent increase in radiotracer activity in the bladder was seen (Fig. 3b). As early as during during second frame (8–16 s), PET renograms demonstrated rapid radiotracer uptake in the renal cortex. Thereafter, a transient increase in radiotracer activity in the cortex, followed by transit to the collecting system was seen in tomographic views (Fig. 3c). Split renal function assessment demonstrated a normal renogram pattern (Fig. 3d). In vivo biodistribution showed favourable results, with the kidneys having the highest radiotracer accumulation, even 60 min after injection. Of note, radiotracer concentrations in the intestines and liver remained stable over time, suggesting low hepatobiliary clearance and exclusive renal secretion of [^18^F]FDS. Post-mortem tissue counting revealed almost identical values to its SPECT counterpart [^99m^Tc]DTPA. The lack of radiolabelled metabolites in the blood and urine 35 min after injection was confirmed by thin-layer radiochromatography. This feasibility study in healthy rats suggested that [^18^F]FDS is freely filtered at the renal glomerulus, and this is in line with previous findings showing rapid clearance of exogenous administered sorbitol that is identical to inulin clearance, as measured in dogs and humans. Again, this may be explained by the underlying sorbitol structure of [^18^F]FDS [14].

Plasma protein binding also has a major impact on radionuclide radiotracer kinetics and is considered a major obstacle to GFR estimation. GFR underestimation might occur, as only the free fraction of the radiotracer is filtered at the glomerulus. Protein binding of [^99m^Tc]DTPA has been reported to range from 2% to 10% [37, 38], whereas [^18^F]FDS demonstrates minimal in-vivo serum protein binding of <0.1% [38].

**Fig. 4:**
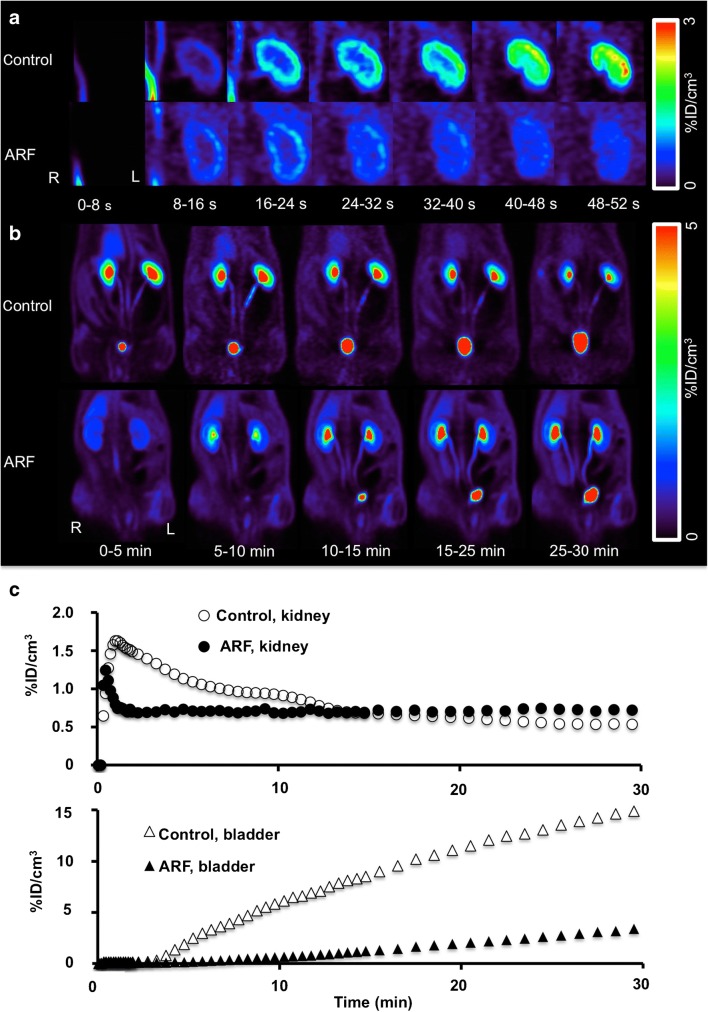
In vivo [^18^F]FDS PET imaging in control rats and acute renal failure (ARF) rats. **a** Dynamic coronal images of the left kidney show rapid tracer uptake in the renal cortex in the control rat, but reduced tracer uptake in the renal cortex in the ARF rat. **b** Whole-body dynamic coronal PET images show high tracer secretion exclusively via the kidneys and a time-dependent increase in bladder activity in the control rat, but reduced renal tracer secretion via the kidneys and a delayed increase in bladder activity in the ARF rat. **c** Average time–activity curves for the kidneys (*top*) and bladder (*bottom*) obtained by dynamic PET imaging indicate low tracer secretion via the kidneys in the ARF rat. Modified from Werner et al. [15]; copyright Society of Nuclear Medicine and Molecular Imaging, Inc.

**Fig. 5:**
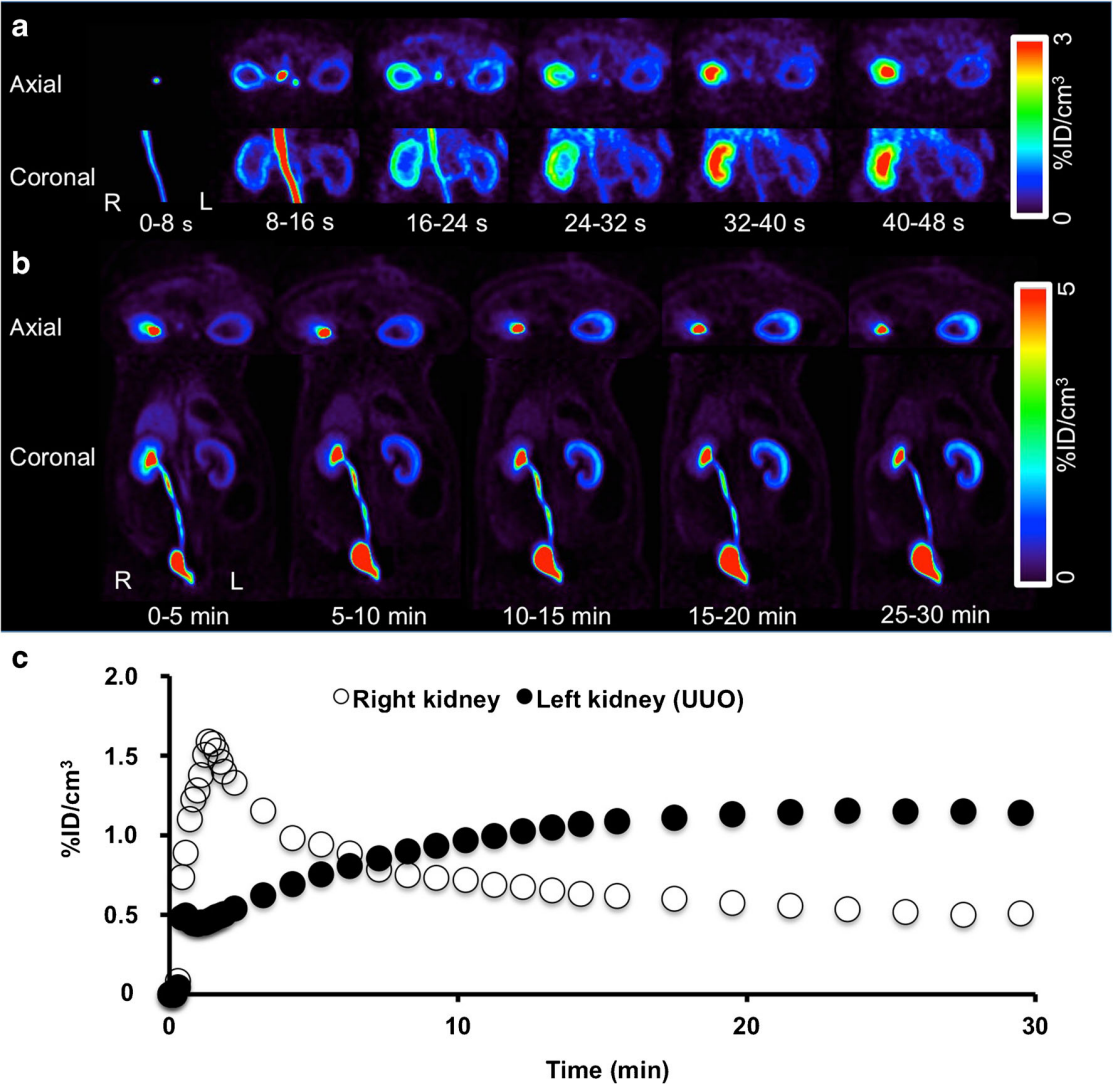
In vivo [^18^F]FDS PET imaging in rats with unilateral ureteral obstruction (UUO). **a** Dynamic coronal images show reduced tracer uptake in the renal cortex of the UUO kidney, but rapid tracer uptake in the renal cortex of the contralateral kidney. **b** Whole-body dynamic axial and coronal PET images show no excretion of [^18^F]FDS into the renal pelvis from the UUO kidney, but excretion of [^18^F]FDS into the renal pelvis from the contralateral kidney at 0–5 min. A time-dependent increase in renal uptake can be seen in the UUO kidney. **c** Average time–activity curves for the kidneys obtained by dynamic PET imaging indicate tracer deposition in the renal cortex of the UUO kidney. Modified from Werner et al. [15]; copyright Society of Nuclear Medicine and Molecular Imaging, Inc.

In light of these encouraging findings, two rat models of renal disorders have been investigated to determine the potential clinical benefit of this radiotracer. First, acute renal failure (ARF) was induced by intramuscular injection of glycerol in the rats. Second, unilateral ureteral obstruction (UUO) was obtained by complete ligation of the left ureter near the renal pelvis. While healthy control rats showed a normal distribution pattern, ARF rats showed a significantly reduced uptake in the renal cortex along with relatively low excretion through the urinary collecting system (Fig. 4a, b). Renograms showed a nonfunctioning pattern, with lower radiotracer secretion via the kidneys in ARF rats than in healthy control rats (Fig. 4c). On the other hand, UUO rats demonstrated significantly delayed uptake on the obstructed left side, with no transit into the collecting system. This observation was in contrast to the contralateral nonaffected kidney that demonstrated normal distribution of [^18^F]FDS (Fig. 5a, b). Renograms showed a typical obstructed pattern, with no further peak during the parenchymal phase and progressive parenchymal accumulation (Fig. 5c) [15].

**Fig. 6:**
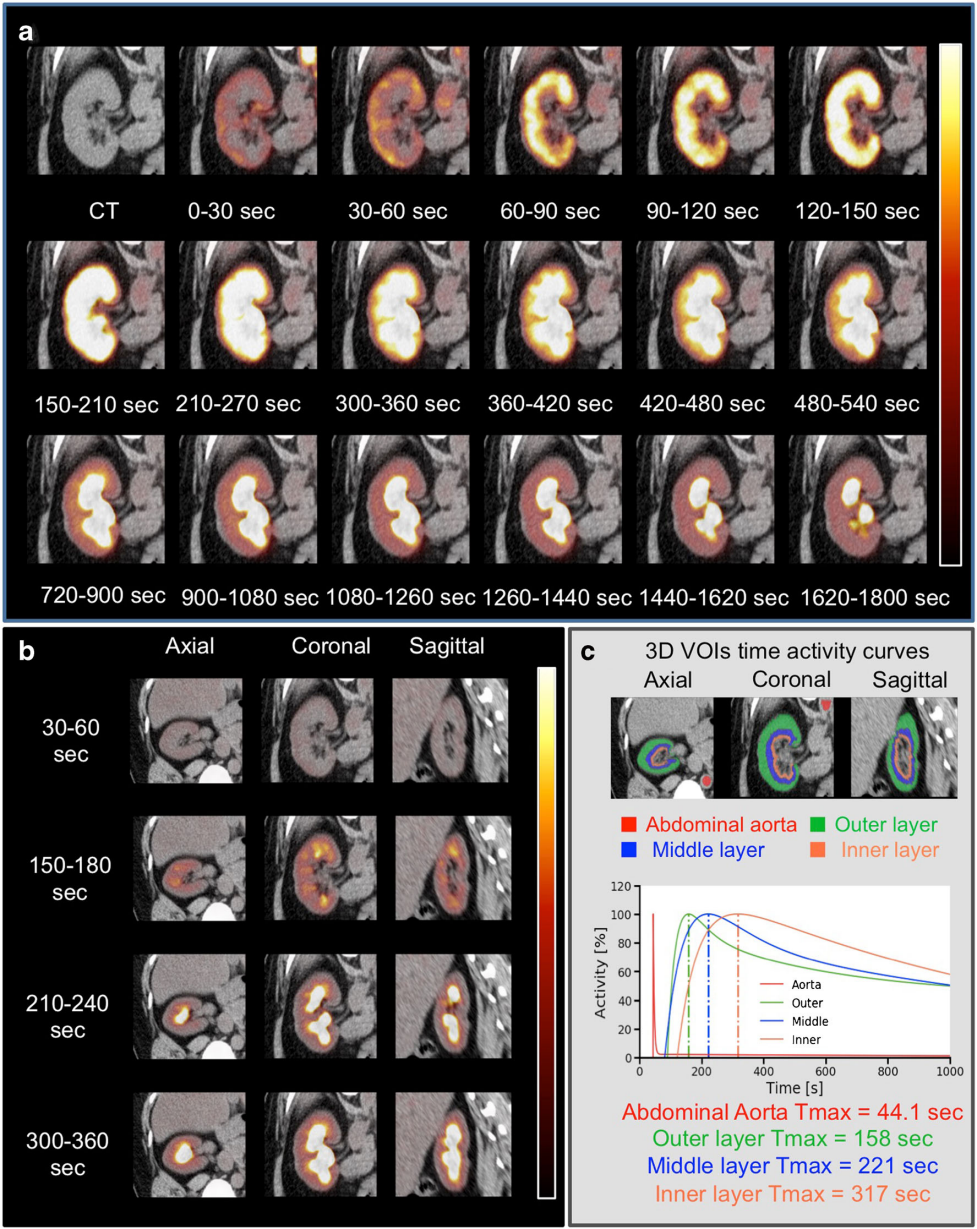
[^18^F]FDS PET/CT imaging of the right kidney in a 48-year old female volunteer. Dynamic coronal images (**a**) and axial, coronal and sagittal images (**b**) show rapid radiotracer accumulation in the renal cortex, followed by radiotracer excretion. **c** Average time–activity curves obtained by dynamic PET imaging. Three-dimensional (3D) volumes of interest (VOIs) were placed on the outer layer (corresponding to the cortex, *green*) as well as on the middle layer (*blue*) and inner layer (*orange*) corresponding to the medulla. Modified from Werner et al. [40]

In the first study in humans investigating the use of [^18^F]FDS, dynamic [^18^F]FDS PET was performed in two healthy volunteers. The radiotracer was shown to pass through the renal parenchyma and transit into the collecting system. The radiotracer gradually increased in the renal parenchyma up to 60 s after administration (i.e. blood flow) and was then excreted. Derived split functional renograms demonstrated a normal pattern in both volunteers and comprised blood flow, parenchymal and excretory phases (Fig. 6a, b). Volumes of interest covering the renal cortex as well as the renal medulla confirmed successive transit of the radiotracer through the kidneys (Fig. 6c). The derived maximal parenchymal activity (*T*_max_, 3 min after injection) is in line with findings for [^99m^Tc]DTPA and [^99m^Tc]MAG3 [39]. No adverse effects due to [^18^F]FDS administration were reported [40].

### Re(CO)_3_([^18^F]FEDA)

In contrast to [^68^Ga]EDTA and [^18^F]FDS that are mainly used for the assessment of GFR, efforts in recent years have focused on developing and investigating other renal PET probes that reflect tubular function and ERPF, e.g. Re(CO)_3_([^18^F]FEDA) and its SPECT analogue [^99m^Tc](CO)_3_(FEDA) [3]. [^99m^Tc](CO)_3_(FEDA) shows rapid renal excretion similar to that of [^131^I]orthoidodohippurate ([^131^I]OIH) in rats [16, 41]. Based on these encouraging results, the authors demonstrated similar findings by developing an efficient, one-step radiosynthesis of an ^18^F-Re-tricarbonyl PET tracer, namely Re(CO)_3_([^18^F]FEDA). Re(CO)_3_([^18^F]FEDA) shows high renal specificity, high in vitro and in vivo stability and rapid renal excretion, which are comparable to those of its analogue [^99m^Tc](CO)_3_(FEDA) [17]. The pharmacokinetic properties of Re(CO)_3_([^18^F]FEDA) are also comparable to those of [^131^I]OIH, which is considered the reference standard for ERPF measurement [17, 42]. As a component of a pair of analogous ^18^F/^99m^Tc renal imaging agents with almost identical kinetic properties, [^99m^Tc](CO)_3_(FEDA) could be available as a kit which is five times cheaper than Re(CO)_3_([^18^F]FEDA). However, if there is a shortage of ^99^Mo that would lead to [^99m^Tc](CO)_3_(FEDA) becoming unavailable, it could be replaced by the analogous PET radiotracer. Such an approach would allow direct comparisons between a previous [^99m^Tc](CO)_3_(FEDA) SPECT study and a subsequent Re(CO)_3_([^18^F]FEDA) PET study [17].

### Al[^18^F]NODA-butyric acid

Biodistribution studies in normal rats and rats with simulated renal failure (by ligation of the renal pedicles) have revealed that Al[^18^F]NODA-butyric acid is exclusively secreted through the renal system. Thus, this radiotracer may also provide reliable estimates of ERPF [43].

### *p*-[^18^F]Fluorohippurate ([^18^F]PFH)

Because of its similar structure to *p*-aminohippurate, which is considered the gold standard for ERPF measurement, [^18^F]PFH was identified by Awasthi et al. as a potential renal PET imaging agent [44]. Pathuri et al. [45] investigated this compound in healthy rats and compared the renogram patterns with those obtained with the gold standards [^125^I]OIH and [^99m^Tc]MAG3. Notably, compared with the derived renogram parameters (*T*_max_, *T*_1/2max_) obtained with [^99m^Tc]MAG3, the parameters obtained with [^18^F]PFH were closer to those obtained with [^125^I]OIH, and [^18^F]PFH also provided better image quality [45]. Another study in Han:SPRD rats with slowly progressive autosomal dominant polycystic kidney disease showed that [^18^F]PFH could be a surrogate marker for disease progression, which further emphasizes the potential clinical utility of this agent in future translational approaches [46].

### [^18^F]FDG

[^18^F]FDG is involved in multiple physiological processes and thus may not be an ideal PET agent to assess renal function. However, as [^18^F]FDG is also considered the “workhorse” in nuclear oncology imaging, it would be of great benefit if basic kidney parameters could be derived from a routine [^18^F]FDG scan. In a study by Geist et al. [47], 24 healthy volunteers underwent [^18^F]FDG dynamic PET/magnetic resonance imaging, and Patlak analysis was performed to determine GFR and ERPF. These quantitative indices correlated with both [^99m^Tc]MAG3 tubular extraction rate and blood-based creatinine clearance in an acceptable range (*R* = 0.73–0.78).

Table 1 summarizes the key properties and limitations of the ^68^Ga-labelled and ^18^F-labelled PET radiotracers reviewed.

**Table 1:** Comparison of ^68^Ga-labelled and ^18^F-labelled PET tracers for renal function assessment

Labelling	Renal PET radiotracer	Reflecting	Uptake mechanism/filtration	Advantages	Limitations
^68^Ga	[^68^Ga]EDTA ([^68^Ga]ethylenediaminetetraacetic acid)	GFR	Filtered at the glomerulus	Longest experience in clinical practice [6] Amount of administered activity lower than with [^99m^Tc]DTPA Excellent correlation with the gold standard [^51^Cr]EDTA [13] Split renal function assessment comparable to that with [^99m^Tc]DTPA (with advantage of imaging in the same study) [6]	Cost-effectiveness data on a larger scale are lacking Limited to university hospitals/tertiary referral hospitals
	[^68^Ga]DTPA ([^68^Ga]diethylenetriamine-pentaacetic acid)		Filtered at the glomerulus		Marked underestimation of GFR of up to 80% compared to [^68^Ga]EDTA [23]
	[^68^Ga]NOTA (1,4,7-triazacyclononane-1,4,7-triacetic acid)		n/a	Low binding to serum proteins and red blood cells Comparable GFR values to [^51^Cr]EDTA in mice Easy to prepare [24]	Assessments for renal PET imaging in humans are needed
	[^68^Ga]IRDye800-tilmanocept		Receptor binding to glomerular mesangial cells [27]	Receptor-based imaging biomarker to monitor progression of glomerular diseases, in particular diabetic nephropathy [27]	Assessments for renal PET imaging in humans are needed
^18^F	[^18^F]FDS (2-deoxy-2-[^18^F]fluorosorbitol)	GFR	Freely filtered at the glomerulus [14]	Simple one-step reduction of the most frequent PET radiotracer [^18^F]FDG [35] Extensively tested in a preclinical setting [14, 15] First study in humans showed no adverse events [40]	Cost-effectiveness data are lacking No data available regarding potential benefit over conventional scintigraphy agents
	Re(CO)3([^18^F]FEDA) ([^18^F]Re(CO)_3_-*N*-(fluoroethyl)iminodiacetic acid)	ERPF	Organic anion transporter 1 [17]	High renal specificity and rapid renal tracer excretion, similar to that of [^131^I]OIH [16, 17] Pair of analogous tracers: if shortage of ^99^Mo occurs, Re(CO)_3_([^18^F]FEDA) would still allow direct comparison with previous [^99m^Tc](CO)_3_(FEDA) studies [17]	Cost-effectiveness data are lacking Assessments for renal PET imaging in humans are needed
	Al[^18^F]NODA-butyric acid		Unknown [43]	Biodistribution studies in rats demonstrated exclusive secretion via the renal system [43]	Assessments for renal PET imaging in humans are needed
	[^18^F]PFH (*p*-[^18^F]fluorohippurate)		Organic anion transporter 1 [45]	Similar structure to the gold standard for ERPF measurement (*p*-aminohippurate) [44] Could serve as a surrogate marker in polycystic kidney disease [46]	Assessments for renal PET imaging in humans are needed
	[^18^F]FDG (2-deoxy-2-[^18^F]fluoro-d-glucose)	GFR and ERPF	Filtered at the glomerulus, partially reabsorbed in the proximal tubule [47]	Derived GFR/ERPF from Patlak analysis demonstrated acceptable correlation with [‘^99m^Tc]MAG3 tubular extraction rate [47] Basic kidney parameters could be derived from a routine oncology PET scan [47]	[^18^F]FDG is involved in multiple physiological processes and thus may not be an ideal renal PET radiotracer [47]

*GFR* glomerular filtration rate, *ERPF* effective renal plasma flow, *[*^*131*^*I]OIH* [^131^I]orthoidodohippurate, *[*^*99m*^*Tc]MAG3* [^99m^Tc]mercaptoacetyltriglycine

## Clinical indications for renal PET imaging

PET offers several advantages over conventional scintigraphy, although the high costs of PET studies are a consideration in deciding the extent to which such PET radiotracers can be employed in clinical routine. In addition, another major obstacle in using renal PET imaging in humans has to be addressed: current PET cameras may not allow imaging of the entire urinary system in a single field of view. Barring the purchase of longer-bore scanners specifically for renal functional imaging evaluation, a solution to this problem is multi-bed-position PET acquisitions, which may adversely affect diagnostic accuracy [13, 15]. In this regard, one may speculate as to the pathophysiological conditions that would justify the use of such an expensive and complex noninvasive metric to measure renal function [3]. [^68^Ga]EDTA may be ideal for monitoring haemodynamically significant renal artery stenosis, as the short half-life of ^68^Ga allows completion of a captopril renal study in a single day [6, 48]. Blaufox and others have suggested that monitoring renal function during chemotherapy and assessment of split renal function (e.g. prior to living kidney donation or radiation therapy) may be suitable indications for renal PET imaging [3, 49–51]. Notably, common scintigraphy/SPECT approaches may lead to underestimation of the relative functional performance of one of the kidneys (e.g. caused by malrotation), which may have a significant impact on the eligibility of a living donor for donating a kidney for transplantation [52, 53]. Hybrid PET/CT to assess renal function including the most modern multislice CT scanners for anatomical coregistration may be helpful to guide the treating urologist or nephrologist in identifying appropriate donor candidates [3].

Apart from these considerations, theranostic approaches to the treatment of neuroendocrine tumours (NET) using [^68^Ga]DOTA-D-Phe-Tyr3-octreotate/octreotide ([^68^Ga]DOTA-TATE/TOC) PET and [^177^Lu]DOTA-TATE/TOC are increasingly being used, in particular due to the encouraging results of a recent randomized, controlled trial in midgut NET [54]. However, radiolabelled somatostatin analogues can cause a decline in renal function and it has been hypothesized that [^99m^Tc]MAG3 might be a suitable means of evaluating early stages of renal deterioration in patients who have undergone repeated cycles of endoradiotherapy. Although radiolabelled somatostatin analogues most likely provoke a cross-fire effect from adjacent tubules, the tubular extraction rate measured by [^99m^Tc]MAG3 could not identify high-risk patients with a late onset of renal failure [12]. Thus, the PET agents reviewed here for ERPF assessment, including Re(CO)_3_([^18^F]FEDA) and [^18^F]PFH, may be better surrogate markers for identifying patients most at risk. This may also apply to other endoradiotherapies with a potential nephrotoxic profile, e.g. in patients with haematopoietic malignancies treated with the CXC-chemokine receptor 4 ligand [^177^Lu]/ [^90^Y]pentixather or in prostate cancer patients scheduled for prostate-specific membrane antigen to enable beta particle therapy [55, 56].

However, we and others see the most relevant indication of renal PET/CT in paediatric patients [3, 6, 14]. Creatinine clearance-based GFR estimates are routinely performed in children, but variability in body mass limit their reliability, and accuracy is also altered in those with renal and urological disorders [3]. In addition, renal anatomical abnormalities are frequently observed in toddlers and younger adults (e.g. ureteropelvic junction obstruction) [57]. Hence, renal PET may open the door to effective decision making in paediatric patients, as it allows simultaneous assessment of renal function and anatomical coregistration in a single study. Additionally, count rates with PET are higher than with conventional scintigraphy, and thus a much lower activities can be administered [6].

## Conclusion

In recent years, a shift from single-photon-emitting to PET radiotracers has occurred in a variety of clinical settings [58–60], and thus the concept of PET has also been applied to renal radionuclide imaging. In this regard, several novel PET radiotracers for the assessment of renal function are currently emerging: the GFR-reflecting PET probes [^68^Ga]EDTA, [^68^Ga]IRDye800-tilmanocept and [^18^F]FDS, and the tubular agent Re(CO)_3_([^18^F]FEDA) [6, 13–15, 17, 27]. [^68^Ga]EDTA is the only agent to date that has already been evaluated in a large clinical trial [13]. [^68^Ga]IRDye800-tilmanocept shows receptor-mediated binding to glomerular mesangial cells, which in turn may allow monitoring of progression of diabetic nephropathy [27]. In contrast to ^68^Ga-labelled radiotracers, [^18^F]FDS has all the advantages of an ^18^F-labelled radionuclide, such as lower positron energy with higher positron yield and longer physical half-life, which allows distribution by commercial vendors [29, 32]. Apart from these GFR-estimating radiotracers, the analogous pair [^99m^Tc](CO)_3_(FEDA)/Re(CO)_3_([^18^F]FEDA) reflects ERPF and both tubular agents are comparable to the radiotracer reference standard for ERPF assessment, [^131^I]OIH [17, 41].

Renal PET may have incremental value in challenging clinical situations and could provide effective decision support, in particular in paediatric patients. Further research exploring the potential benefit over conventional scintigraphy/SPECT agents and larger clinical trials to identify the most suitable clinical indications and scan timing for each renal PET radiotracer are warranted. Efforts should also turn toward synthesizing novel (SPECT or PET) renal radiotracers that have ideal properties for renal functional imaging, e.g. exclusive kidney extraction and excretion, low plasma protein binding, high metabolic stability, low hepatobiliary clearance, global availability and validated, scan-derived quantitative indices.
